# Gender in the time of COVID-19: Evaluating national leadership and COVID-19 fatalities

**DOI:** 10.1371/journal.pone.0244531

**Published:** 2020-12-31

**Authors:** Leah C. Windsor, Gina Yannitell Reinhardt, Alistair J. Windsor, Robert Ostergard, Susan Allen, Courtney Burns, Jarod Giger, Reed Wood

**Affiliations:** 1 Department of Political Science, Faculty Affiliate, Institute for Intelligent Systems, The University of Memphis, Memphis, Tennessee, United States of America; 2 Department of Government, University of Essex, Colchester, Essex, United Kingdom; 3 Department of Mathematical Sciences, Institute for Intelligent Systems, The University of Memphis, Memphis, Tennessee, United States of America; 4 Department of Political Science, University of Nevada, Reno, Nevada, United States of America; 5 Department of Political Science, The University of Mississippi, Oxford, Mississippi, United States of America; 6 Department of Political Science, Bucknell University, Lewisburg, Pennsylvania, United States of America; 7 College of Social Work, University of Kentucky, Lexington, Kentucky, United States of America; Ghana Health Services, GHANA

## Abstract

In this paper we explore whether countries led by women have fared better during the COVID-19 pandemic than those led by men. Media and public health officials have lauded the perceived gender-related influence on policies and strategies for reducing the deleterious effects of the pandemic. We examine this proposition by analyzing COVID-19-related deaths globally across countries led by men and women. While we find some limited support for lower reported fatality rates in countries led by women, they are not statistically significant. Country cultural values offer more substantive explanation for COVID-19 outcomes. We offer several potential explanations for the pervasive perception that countries led by women have fared better during the pandemic, including data selection bias and Western media bias that amplified the successes of women leaders in OECD countries.

## Introduction

Since the start of the Covid-19pandemic, many have suggested that countries led by women have fared better than those led by men. This popular narrative has appeared in the *New York Times*, *Forbes*, *Vox*, *the Harvard Business Review*, *Stanford Medicine*, and *NBC News* [1–5]. For example, New Zealand’s Prime Minister Jacinda Ardern’s success in “flattening the curve” attracted initial attention and speculation about the role of leader gender in mitigating the deleterious effects of the pandemic. Iceland has garnered similar praise. Recently released scholarly analyses also suggest countries led by women have six times fewer deaths than those led by men [6].

This narrative reflects the political gender double bind, and assigns women leaders traits such as good listening skills, the tendency to seek input and counsel for major decisions, the ability to provide a big-picture overview of a situation, and proficiency in risk management [7, 8]. These traits would arguably make anyone better at managing crises such as the Covid-19 pandemic, and these traits are more commonly proscribed to women than in men [1]. The perspective that women have been better leaders during the pandemic is rooted in selection bias, based on the selective reporting of cases where women-led countries have succeeded in pandemic management, and are focused on OECD countries [9]. These reports fail to acknowledge men-led countries that have done similarly well, while instead emphasizing carefully selected cases where men have not performed well. In this paper we interrogate this narrative, and examine the mechanisms underpinning the outcomes we see. We ask: *Are women better leaders than men during times of crisis*?

Though acknowledging sound reasoning behind the narrative based on previous scholarship on women’s leadership and policy priorities, we argue that the logic behind the narrative is overly simplistic, failing to account for the underlying factors that bring women to national leadership in the first place. Instead, we contend that women are able to attain national leadership positions in countries where core cultural values reward traits often found in women leaders, such a long-term orientation, a collectivist (rather than individualist) focus, and fewer power disparities in society [10]. These countries therefore have policy landscapes that enable leaders to consult with others and carefully weigh options, examine the larger policy/outcome picture of major decisions, and manage risk effectively. Women who lead these countries are able to successfully manage crises like the pandemic not because they are women, but because they are leading countries more likely to elect women to the highest executive office in the first place, and because those countries have policy landscapes and priorities that pre-dispose them to manage risk better.

We examine national-level data for 175 countries around the world, accounting for Covid-19 infection and death rates, cultural traits [10, 11], gender parity in the national assembly, and whether each country is led by a woman or a man. In support of our argument, we find no statistically significant differences between Covid-19 fatality rates in countries run by women versus men unless we account for cultural factors. Specifically, we reveal that having a woman leader does not make a country fare better during the pandemic unless that country also has the cultural values that support female leadership. Given a position of leadership, women leaders are then better able to capitalize on particular cultural values than men, and more likely to turn those values into pandemic management successes than men leaders, while countries without those values fare worse, regardless of whether they are led by men or women.

We note at least three contributions of our work. First, our findings suggest that current analyses that show women-led countries are faring better may suffer from selection bias and/or economic development bias. New Zealand and Iceland, two countries with female leaders lauded for their pandemic management, are small remote islands, and are also OECD countries. By analyzing only an economically advanced subset of cases, other researchers and news outlets amplify a message that does not resonate with the reality of global data. We examine a global sample of leaders, rather than a limited OECD-only analysis, and find that while some women-led countries are experiencing better social outcomes than some men-led countries, the trend is far from universally true.

Second, our consideration of cultural factors as underpinning mechanisms of national-level women leadership enables us to suggest *why* we do not see worldwide differences in pandemic management success according to leadership gender. Our findings speak to important connections between national cultural values, the management structures they allow, and the types of priorities, policies, and leaders they produce in society. The sample of women who lead countries and who have real executive power is quite small as compared to the number of men-led countries in the world. Yet the cultural values that undergird women’s leadership in many cases would also produce more beneficial Covid-19-related outcomes if the country were led by a man.

Our work suggests that leader gender does matter, but not necessarily in the ways highlighted by the current discussion. Public attention has focused on female chief executives, rather than the types of society-wide values and priorities that contextualize their leadership. Rather, we note that women-led countries are positioned to excel in many ways after the pandemic because of gendered policymaking incentives embodied in the national culture. Our work therefore extends the public discussion and prompts future investigations into important related questions regarding how women-led countries might fare better in the long term in ways such as economic recovery, unemployment, and poverty alleviation. We find that the interaction between having a woman as a country leader and having certain country-level cultural features that encourage provision of public goods also provide more protection against dying from Covid-19.

Below we explore the theoretical foundations for public perception for why women-led countries have outpaced their male counterparts in the pandemic policy arena. We test our expectations using data from several sources, providing robustness checks with multiple methodological approaches. We conclude with a discussion of the big-picture implications of our findings in the context of the current pandemic and beyond.

## Gendered perceptions of pandemic success

The prevailing narrative that women leaders are doing better at managing the pandemic is based largely on the idea of the *political double bind* [12, 13]. Beholden to gender stereotypes and scrutiny that men in power escape, women leaders must be both stereotypically masculine (i.e., “act like a leader”) and stereotypically feminine (i.e., “act like a woman”). This double bind hinders women leaders’ career aspirations because “acting like a leader” makes a woman too masculine to still be able to “act like a woman”. Without any option but to violate at least one of these stereotypes, many women end up violating both at once, with their constituents likely to punish them for it [14, 15].

Yet during a pandemic, the duality of expectations in the double bind actually positions women to thrive in their capacity as leaders because pandemics are a situation that demand assertive action and decisiveness (seen as masculine traits) about health and human security (seen as feminine policy areas). Previous scholarship points to at least 4 reasons why women should fare better than men at managing pandemics. First, feminine gendered traits, such as being caring and nurturing, being trustworthy [16, 17], focusing on health, human security, education, and capacity-building [18, 19], and being better at anticipatory policymaking that increases social buffers [20], are appealing to the western world during health crises. Norway’s Prime Minister Erna Solberg exemplifies these qualities. In mid-March, she held a special 30-minute press conference for children to help them process the challenges of the pandemic, telling them, “it’s okay to feel scared [21].” Second, women’s priorities are categorized as communal and grounded in prioritizing preventive measures [22, 23], factors consistent with high levels of disaster preparedness. Third, women’s leadership is transformational in that it builds capacity and resilience [24], ideal for withstanding shocks like pandemics. Pandemics offer women leaders the opportunity to demonstrate their capacity to navigate the double bind by conforming to the gendered norms of being compassionate, and also demonstrating their ability to be strong leaders in enacting tough policies such as closing borders and mandating lockdown measures, as Jacinda Ardern of New Zealand did.

Men’s leadership, on the other hand, is categorized as transactional, providing rewards for individual behavior and waiting for problems to innovate solutions [20, 25], which is consistent with waiting until disaster strikes to think about relief measures. Policy interventions such as stimulus payments to citizens, are post-hoc interventions to alleviate the economic impacts of the pandemic [26]. Finally, during a crisis, citizens are more willing to follow the advice of leaders they trust [27], so women leaders should have an easier time managing crises like pandemics. The complexities of a global pandemic are therefore ideal for highlighting women’s leadership and policymaking assets.

Additionally, women leaders’ compassionate, relatable communication styles make the public feel more secure about the future. Policy speeches given by women world leaders in mid-March 2020 helped shape global perception that women chief executives were controlling the spread of the virus better than men [28]. These leaders have utilized familiar, prosocial, feminine frames in their speeches that align with the political double bind and resonate with citizens [29]. As Windsor et al., note, women leaders’ speeches have also been more cohesive and more likely to have social and community themes than those of male leaders [28]. Combined with select cases where women-led countries have successfully flattened their curves, these observations have contributed to a blanket generalization that women-led countries have fared better than men-led countries during the pandemic.

### Interrogating the current narrative of women leaders’ Covid success

**Fig 1:**
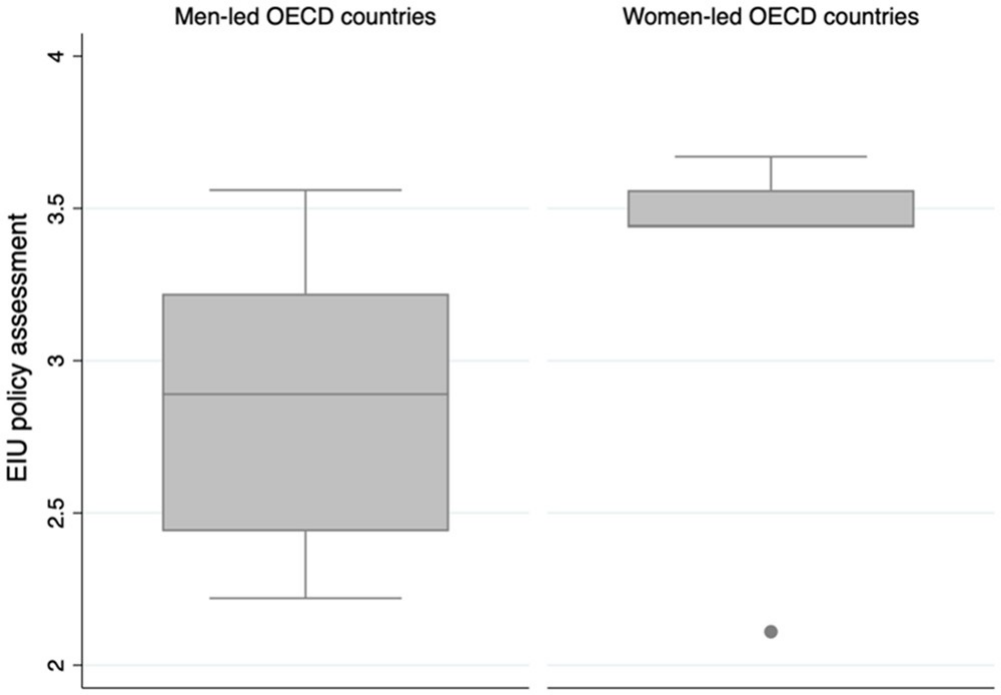
Quality of Covid-19 policy responses from the Economist Intelligence Unit assessment of OECD countries, graphed according to gender of the national executive (graph generated by authors).

Though compelling based on the scholarship cited above, emerging evidence suggests that the narrative of women being better leaders during the pandemic demands closer empirical interrogation. First, consider Economist Intelligence Unit rankings of OECD countries by the quality of their Covid-19 responses, where higher values indicate better policy responses [30]. We graph these according to the gender of the national executive (Fig 1). The graph certainly suggests that women-led countries are over-represented among those with the highest rankings, and consistent with early research on this topic [6]. What this ranking (and research) do not capture, however, is the full global landscape of countries. Rather, they reinforce Western biases and perceptions of gender and leadership responses to the pandemic, ignoring the responses of non-OECD countries entirely. A country such as Vietnam, for example, a man-led country with 97 million people and land border with China that to date has barely 1000 Covid-19 cases and fewer than 40 deaths [31], fails to enter the picture.

By focusing only on OECD countries where democracies are over-represented, we also fail to consider pandemic management across varied regime types. While democracies and highly developed countries tend to over-provide public goods, dictatorships may have an “authoritarian advantage” in implementing comprehensive pandemic policies that limit the social, political, medical, and economic impacts of the virus. Combined with lower levels of economic development, the authoritarian advantage becomes particularly beneficial pandemic-wise in agrarian societies where people already live farther apart than they would in an urban/industrial context [32, 33].

Key to our argument, the type of regime and governance system may influence the leader’s and country’s overall response. Freedom House classifies countries along three categories: Free; Partly Free; and Not Free [34]. Freedom House provides annual country-level ratings related to political rights and civil liberties such as respect for the rule of law, political pluralism, the functioning of government, and electoral processes in countries and territories throughout the world. Democracies—free countries—have an incentive to provide public goods that buffer against pandemics and other natural disasters, given that leaders whose policies fail their constituents during times of crisis face almost certain removal come re-election time [34, 35]. Leaders in authoritarian countries, on the other hand, also behave differently with respect representation, given their means for remaining in office differ from those in democracies. Democracies (free countries) also tend to produce more women chief executives than non-democracies (not free countries), as well. If authoritarian (not free) regimes are more likely to manage pandemics according to different incentives, and to promote women in leadership positions for reasons different from free countries, we must include them in any empirical analysis of gender, leadership, and pandemic outcomes.

#### The importance of preparation

**Fig 2:**
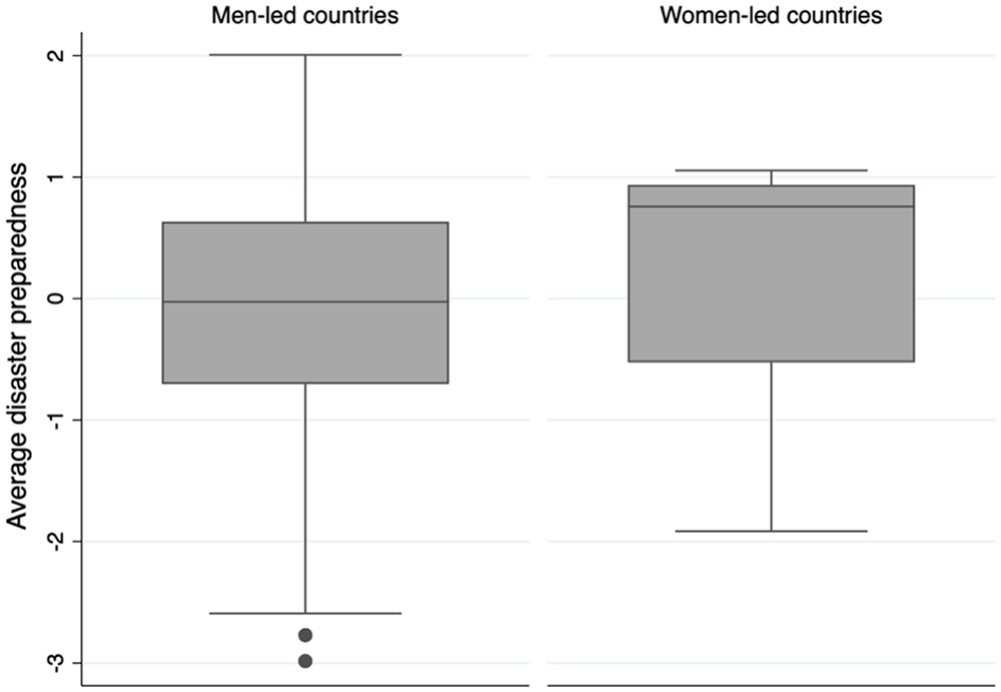
Disaster preparedness in men- and women-led countries, calculated by authors based on Hyogo Framework for Action [38].

Second, critical to this debate is the fact that disaster outcomes are not merely dependent on leadership now, but also on preparation that took place previously [36, 37]. Strong disaster preparedness systems would have protocols in place for confronting threats such as a pandemic that today’s leaders need only activate, adapt, and implement, meaning that women leaders today will be better placed to manage pandemics if they can rely on preparedness and policies made by leaders in the past. Consider another perspective from the disaster preparedness framework. To gauge general preparedness we calculate standardized average disaster preparedness scores according to the *Hyogo Framework for Action* [38], and graph this by current leader gender as shown in Fig 2. This snapshot of pandemic management and leadership gender does suggest that women-led countries have higher average preparedness ratings than men-led countries.

A surface-level interpretation of the graph might suggest that preparedness levels depend on leader gender, and that women lead countries that are more prepared because of the woman’s leadership. But the timing of disaster preparation as, by definition, occurring before a critical event like a pandemic suggests that the relationship between disaster preparedness and leader gender is more nuanced—that in fact, some countries are more likely both to be prepared for disaster *and also* to elect women to leadership in government. We posit that the common factor among these countries is cultural.

#### Feminine social cultural norms

Third, cultural norms, beliefs, and behaviors can be classified according to similar attributes across countries. Hofstede [10, 11] categorizes 91 countries according to 6 specific dimensions: power distance, uncertainty avoidance, individualism versus collectivism, masculinity versus femininity, long-term versus short-term normative orientation, and indulgence versus restraint. The literature about these cultural features suggests that women-led countries should have overall better responses to the COVID-19 pandemic because they align with policies that advance communal well-being. From a theoretical perspective, people in countries with women chief executives and greater women’s representation in the legislature may have better social and physical outcomes—fewer deaths—during a pandemic both because of the emphasis on baseline preparation as a function of their caregiver role expectations, and also because they can demonstrate masculine leadership by acting decisively to close borders and implement other emergency executive measures unilaterally. In other words, women can both react compassionately by asking their constituents to behave in accordance with promoting the common good, and also aggressively by closing down borders. These actions fulfill both gendered traits, and work in the favor of women leaders.

The cultural factor most relevant to our inquiry is the masculinity versus femininity dimension [39]. Countries that elevate feminine traits prioritize having minimal role differentiation between genders, encourage sympathy for the weak, and elect women to multiple political positions [10, 40]. Countries that prioritize feminine cultural norms should also have less to reconcile with regards to the political double bind [41]. To that end, if a woman currently leads a country that embodies feminine cultural norms, then by definition there is society-wide support for policies that would benefit the public good. In turn, the woman leader should have more flexibility in the policies she can enact, which becomes especially relevant in managing a pandemic. In more feminine societies, women should be rewarded for preventive and interventive policies that elevate their country’s baseline resilience from pandemics, limit the immediate pandemic-related damage, and mitigate against long-term negative consequences. Other cultural features align with feminine social norms. These include less power distance (more egalitarianism), less uncertainty avoidance, more collectivism, longer-term orientation, and more indulgence (e.g., basic needs satisfied; self-fulfillment needs are met) [42].

**Fig 3:**
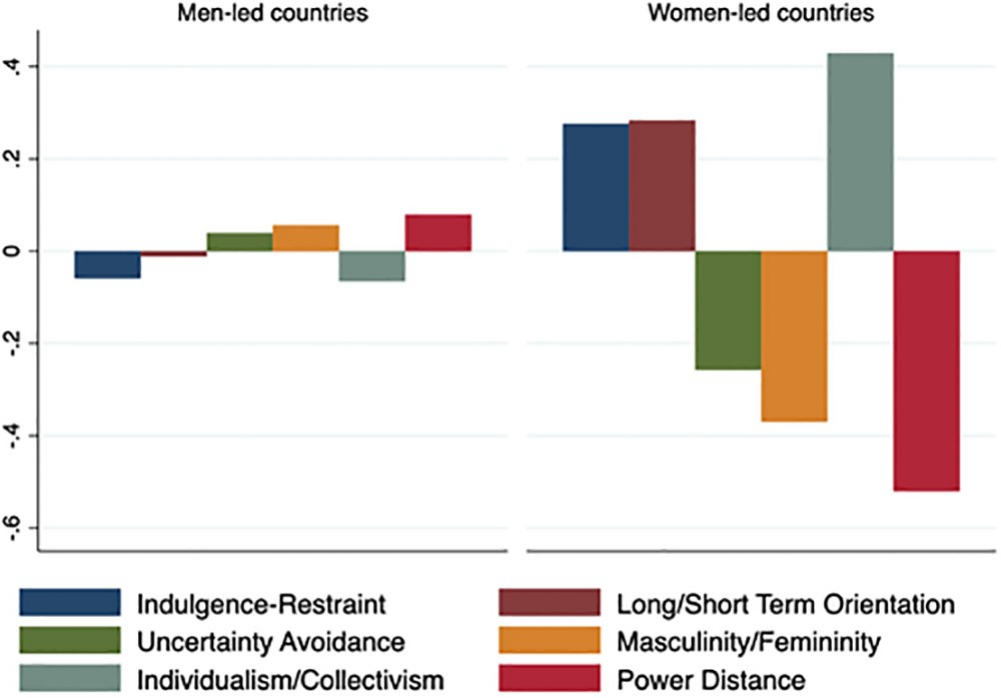
Differences in cultural dimensions between countries led by men and women, using Hofstede [10, 11] cultural data and authors’ definition of leadership gender. N = 175; n_women-led_ = 16; n_men-led_ = 159.

In Fig 3 we graph Hofstede’s 6 cultural dimensions for 175 countries according to whether the country is led by a man or a woman, defining a country as “woman-led” if a woman holds executive authority in that she commands a military, which we provide subsequently in Table 3. We have standardized these features at the mean (0), so that the height of a bar represents distance from the mean. Bars that extend above the mean show higher than average values for a particular dimension, and bars that extend below the mean show lower than average values for a dimension.

Across each of these cultural dimensions, countries led by men and women show descriptive differences. For the indulgence/restraint dimension (the dark blue bars), women-led countries exhibit more indulgence than the average, and men-led countries exhibit less. Similarly, women-led countries exhibit higher-than-average long-term orientation and individualism (brown and sage bars), while men-led countries exhibit lower-than-average values of the same. Meanwhile, women-led countries are lower than the average in terms of tendency to avoid uncertainty (green), masculinity (yellow), and power distance (red), while men-led countries are the opposite. Across all aspects, women-led countries exhibit larger distances from the mean than men-led countries. We expect that a particular combination of cultural traits would support better outcomes during a pandemic regardless of whether or not the country were led by a man or woman. The include: more indulgence; less uncertainty avoidance; more collectivism; more long-term orientation; more femininity; and less power distance in society.

#### Gender and pandemic policies

Given what we know about disaster management, we can see how these individual cultural aspects could provide protection against poor pandemic outcomes. For example, disasters are times of great uncertainty, when individual autonomy must be sacrificed in the process of following government guidance and believing constantly updated information [36, 43].

As with any new virus presentation, the SARS-CoV-2 virus introduced a multitude of unknown factors about its pathology and presentation in patients, uncertainty of quarantine and lockdown timelines and policies, and the complexities of therapies and vaccines that would allow states to return to a degree of normalcy [44, 45]. Successful pandemic management thus depends on the ability to mitigate that uncertainty and to adapt strategies and behaviors consistently updated with new information, rather than forcing a single course of action for the sake of constancy. It is not surprising that Erman and Medeiros find that cultures trending uncertainty avoidance are associated with higher COVID-19 fatalities, both in terms of case fatality and mortality rates [46]. Countries more able to tolerate uncertainty should be both better able to handle pandemics and more likely to elect women leaders.

Erman and Medeiros further find that cultures trending toward individualism and long-term orientation are associated with higher COVID-19 fatalities [46]. We posit that these cultural factors could play a role in both a country’s ability to handle the COVID-19 pandemic, and its election of women. And, when a woman leads a country with traits more conducive to successful pandemic management, we expect them to perform better than when men lead countries with similar cultural traits.

The adoption of public health strategies has presented as a mixed bag associated with a state’s idiosyncratic circumstances more so than any clear gender-specific issues. We obtained representative policy data for 12 of the 15 countries that are female led in our sample from the CoronaNet project. The CoronaNet project tracks covid-19 mitigation policies adopted by governments across 18 specific policy areas. We divided these policies between those that target individual behavior and those that are larger state functions, as shown in Table 1. While the aggregated data we use do not take into account the duration of policies (for instance, a quarantine policy could be put in place for several months, but would only count as a single policy) or the changes in policy specifications, they do show patterns that emphasize government policies broadly and clearly in areas that governments have found easy to regulate or problematic in terms of balancing public health with services and the economy.

**Table 1:** Individual and state function mitigation policies.

Individual Behavior Targeting	State Function Targeting
Curfew	Anti-Disinformation Campaign
Health Monitoring	Closure and Regulation of Schools (Primary/Secondary)
Health Testing	Declaration of Emergency
Hygiene	External Border Restrictions
Lockdown	Health Resources
Public Awareness Measures	Internal Border Restrictions
Quarantine/Self Quarantine	New Task Force or Administrative Configuration
Restrictions on Mass Gatherings	Other (uncategorized policies)
Social Distancing	Restrictions and Regulation of Business
	Restrictions and Regulation of Government Services

As we show in Table 2, we observe very different aggregate policies in countries led by men and women. We use data from the CoronaNet project which tracks state policies implemented during the pandemic. We coded each policy for targeting individual or state level actions. We then calculated them as percentages of total policies implemented by the country. Data were not available for Aruba, Hong Kong, Serbia, and Turks and Caicos Islands. Women-led countries overwhelmingly pursue state-level policies, whereas countries led by men pursue more individual-level policies. We selected the matching men-led countries based on our empirical analysis, discussed below, through nearest-neighbor matching to women-led countries (see Table 6). While all countries have pursued both individual- and state-level approaches to some degree, it is noteworthy that women-led countries have focused on the “big picture” whereas men-led countries have focused on regulating individual behavior. While these policies have not led to statistically significant differences in Covid-19-related fatalities, they have contributed to the perception that women-led countries are doing better given their pursuit of higher-profile macro-level policies.

**Table 2:** Individual- and state-level behavior targeting mitigation policies as a percent of all state policies in women-led and men-led countries.

Women-led countries	Individual-level	State-level	Nearest Neighbor Men-led countries	Individual-level	State-level
Bangladesh	11%	89%	Morocco	60%	40%
Barbados	45%	55%	Uruguay	57%	43%
Belgium	53%	47%	Sweden	54%	46%
Bolivia	19%	81%	Mexico	73%	27%
Denmark	21%	79%	Netherlands	82%	18%
Finland	23%	77%	Austria	58%	42%
Germany	25%	75%	United Kingdom	60%	40%
Iceland	58%	42%	DR Congo	50%	50%
Myanmar	44%	56%	Switzerland	83%	17%
New Zealand	24%	76%	Montenegro	75%	25%
Norway	36%	64%	Bhutan	25%	75%
Taiwan	32%	68%			

To test the prevailing narrative that women-led countries have fared better during the pandemic than men-led countries. Motivated by the above theoretical and empirical challenges to this narrative, we develop the following hypotheses: From the preceding discussion we generate the following hypotheses:

H_1_: Countries led by women chief executives will have fewer Covid-19-related deaths.
H_2_: Countries with stronger feminine culture, longer-term orientation, more indulgence, less power distance, greater tolerance for uncertainty, and more collectivism will have fewer Covid-19-related fatalities.
H_3_: Leadership by a woman will amplify the effects of cultural traits that are already beneficial to pandemic management, compared to leadership by a man.

## Data

Data for this analysis comes from Our World in Data [47], the World Bank [48], Freedom House [34], and the World Health Organization [49]. We examine 175 countries, 16 of which we consider woman-led. A fundamental consideration of this examination of women leaders is whether there are sufficient women chief executives to actually test and confirm the narrative. A country is coded as woman-led if a woman holds executive authority and wields real power (in that she commands a military), regardless of whether she is head of state or head of government (see Table 3 which lists all countries in our sample according to gender of the chief executive, and includes the name of the chief executive for countries led by women). We tally 8% of countries led by women chief executives during the pandemic (92% led by men). Countries such as Ethiopia, Nepal, Singapore, and Greece have women in leadership roles with ceremonial, nominal power, and so are coded as being led by men.

**Table 3:** Chief executives according to sex π.

**Countries with Women as Chief Executives**	
**Country Name**	**Chief Executive**
Aruba	Evelyn Wever-Croes
Bangladesh	Sheikh Hasina
Barbados	Mia Mottley
Belgium	Sophie Wilmès
Bolivia	
Denmark	Mette Frederiksen
Finland	Sanna Marin
Germany	Angela Merkel
**Country Name**	**Chief Executive**
Hong Kong	Carrie Lam
Iceland	Katrín Jakobsdóttir
Myanmar	Aung San Suu Kyi
New Zealand	Jacinda Ardern
Norway	Erna Solberg
Serbia	Ana Brnabić
Taiwan	Su Tseng-Chang
Turks and Caicos Islands	
**Countries with Men as Chief Executives**	
Afghanistan	
Albania	
Algeria	
Andorra	
Angola	
Antigua and Barbuda	
Argentina	
Armenia	
Australia	
Austria	
Azerbaijan	
Bahamas	
Bahrain	
Belarus	
Belize	
Benin	
Bermuda	
Bhutan	
Bosnia and Herzegovina	
Botswana	
Brazil	
Brunei	
Bulgaria	
Burkina Faso	
Burundi	
Cambodia	
Cameroon	
Canada	
Cape Verde	
Central African Republic	
Chad	
Chile	
China	
Colombia	
Comoros	
Congo	
Costa Rica	
Cote d’Ivoire	
Croatia	
Cuba	
Cyprus	
Czech Republic	
Democratic Republic of the Congo	
Djibouti	
Dominica	
Dominican Republic	
Ecuador	
Egypt	
El Salvador	
Equatorial Guinea	
Eritrea	
Estonia	
Eswatini	
Ethiopia	
Fiji	
France	
Gabon	
Gambia	
Georgia	
Ghana	
Greece	
Grenada	
Guatemala	
Guinea	
Guinea-Bissau	
Guyana	
Haiti	
Honduras	
Hungary	
India	
Indonesia	
Iran	
Iraq	
Ireland	
Israel	
Italy	
Jamaica	
Japan	
Jordan	
Kazakhstan	
Kenya	
Kiribati	
Kosovo	
Kuwait	
Kyrgyz Republic	
Laos	
Latvia	
Lebanon	
Lesotho	
Liberia	
Libya	
Liechtenstein	
Lithuania	
Luxembourg	
Madagascar	
Malawi	
Malaysia	
Maldives	
Mali	
Malta	
Marshall Islands	
Mauritania	
Mauritius	
Mexico	
Micronesia	
Moldova	
Monaco	
Mongolia	
Montenegro	
Morocco	
Mozambique	
Namibia	
Nauru	
Nepal	
Netherlands	
Nicaragua	
Niger	
Nigeria	
North Korea	
North Macedonia	
Oman	
Pakistan	
Palau	
Panama	
Papua New Guinea	
Paraguay	
Peru	
Philippines	
Poland	
Portugal	
Qatar	
Romania	
Russia	
Rwanda	
Saint Kitts and Nevis	
Saint Lucia	
Saint Vincent and the Grenadines	
Samoa	
San Marino	
Sao Tome and Principe	
Saudi Arabia	
Senegal	
Seychelles	
Sierra Leone	
Singapore	
Slovak Republic	
Slovenia	
Solomon Islands	
Somalia	
South Africa	
South Korea	
Spain	
Sri Lanka	
Sudan	
Suriname	
Sweden	
Switzerland	
Syria	
Tajikistan	
Tanzania	
Thailand	
Timor-Leste	
Togo	
Tonga	
Trinidad and Tobago	
Tunisia	
Turkey	
Turkmenistan	
Tuvalu	
Uganda	
Ukraine	
United Arab Emirates	
United Kingdom	
United States	
Uruguay	
Uzbekistan	
Vanuatu	
Venezuela	
Vietnam	
Western Sahara	
Yemen	
Zambia	
Zimbabwe	

We account for the phased onset of the epidemic across different countries when operationalizing the notion of deaths. For our approach to analyzing Covid-19 death rates, we compute three disparate “starting points”: the day of the first reported case; the day of the first reported death; and the first day that the number of deaths is greater than 1 per million using the BBC criteria [50]. We use these dates to compute three different time scales and for each scale we compute four different measures: deaths per capita 30, 60, 90, and 120 days after the “start”, This leads us to 12 measures on the effectiveness of the national response.

Our two baselines variables are date of first reported Covid-19 case per country, and date of first reported Covid-19 fatality per country. We consider these baselines in case the date of the first reported Covid-19 case is more sensitive to the quality and extent of the countries’ testing regime than the date of the first confirmed Covid-19 fatality. We consider reported Covid-19 deaths at 30, 60, and 90 days after the baseline. Countries without reported Covid-19 deaths are included in the analysis of days since first reported case, but excluded from analysis of days since first reported death, because they have no deaths from which to begin the analysis.

We also include the following covariates from the World Bank in our models: GDP per capita, percent of the population over age 65, total population, total land area, length of land borders, and life expectancy [48]. To operationalize differences in governance, we include Freedom House indicators of Free, Partly Free, and Not Free [34]. To capture elements of culture, we use the six dimensions of culture generated by Hofstede 11). From the Correlates of War project we extract three measures of national borders, by country: total, sea, and land [51].

To investigate women’s leadership more broadly, we also consider the potential of women in national legislative assemblies to affect pandemic outcomes. Research into leadership priorities between women and men in legislative assemblies suggests that more women in legislatures transforms parties and diversifies political agendas [52]. We include data from the International Institute for Democracy and Electoral Assistance data on Women in Parliament from the Inter-Parliamentary Union repository [53].

We have the covariates necessary to perform our first GLM and our matching scheme for 155 male-led and 12 female-led countries. A full list appears in the S1 Appendix. Due to our longer time horizon, far fewer countries drop out now due to not reaching the appropriate point. If we count from the first confirmed case only one country drops out at the 120-day point and that is Lesotho (male-led). If we count from the first confirmed death, then countries drop out as follows: at 30 days: Bhutan, Cambodia, Eritrea, Grenada, Laos, Mongolia, Saint Lucia, Saint Vincent and the Grenadines, Seychelles, Timor (all men-led); at 60 days: additionally, Fiji, Lesotho, Namibia, Papua New Guinea, Uganda, and Vietnam (all men-led); at 90 days: no change from 60 days; and at 120 days: additionally, Central African Republic, Madagascar, Mozambique, Nepal, and Rwanda (all men-led).

If we count from the day that deaths reach 1 per million of population then countries drop out of the analysis as follows: at 30 days: Bhutan, Botswana, Burundi, Cambodia, Eritrea, Grenada, Laos, Mongolia, Mozambique, Papua New Guinea, Rwanda, Saint Lucia, Saint Vincent and the Grenadines, Seychelles, Sri Lanka, Tanzania, Thailand, Timor, Uganda, Vietnam (men-led) and Myanmar (women-led); at 60 days: additionally, Angola, Fiji, Jordan, Lesotho, Namibia, Zimbabwe (all men-led); at 90 days: additionally, Benin, Democratic Republic of Congo, Ethiopia, Gambia, Libya, Madagascar, Malawi, Nepal, Uzbekistan, Venezuela, Zambia. (all men-led); and at 120 days: additionally, Central African Republic, Ivory Coast, Ghana, Guinea, Kenya, Mauritania, Nicaragua, Nigeria, Senegal, Yemen (all men-led).

### Analysis

We include the following covariates: a dichotomous indicator for woman leader; percentage of women in parliament; an indicator for Freedom House status; GDP per capita; percentage of population over 65; land area; land borders; life expectancy; and the six dimensions of culture from Hofstede. Our health indicators are strongly correlated (ρ = 0.74) though the standard variance inflation factors are around 3. We include both since they measure different constructs and these measures have been proposed to address some of the observed national variation in deaths. For example, Italy and Singapore have very similar life expectancy but the Italy has around twice the percentage of the population over 65 and Qatar has a high life expectancy but only 1.5% of its population over 65.

#### Basic differences: T-tests

We perform an initial t-test of the proportion of deaths for the group of countries with female leaders against the group of countries with male leaders, as shown in Table 4. A negative sign means more deaths per capita in women-led countries, and a positive sign means more deaths per capita in men-led countries. As Table 4 shows, we find no statistically significant differences between countries led by men versus women for either reported Covid-19 fatalities, or for Hofstede’s cultural dimensions. While there are no significant differences for culture, we do observe patterns that indicate countries led by women have different cultural practices than those led by men that align with those found by Erman and Medeiros [46]. While their study has an N = 49, this sample is twice that (N = 100). The smaller sample size for models that include culture are a result of the limited number of countries from the Hofstede culture data [11].

**Table 4:** t-test results for number of reported deaths from first case and first death at 30, 60, and 90 days, and for Hofstede cultural dimensions.

		e(b)	t	N
**Covid-19 related deaths**	Case 30d	0.00000257	(-0.2)	186
	Case 60d	-2.36E-06	(-0.09)	186
	Case 90d	-0.0000298	(-0.82)	186
	Case 120d	-0.0000328	(-0.80)	185
	Death 30d	-0.0000159	(-0.79)	174
	Death 60d	-0.0000313	(-0.83)	168
	Death 90d	-0.0000277	(-0.65)	168
	Death 120d	-0.0000187	(-0.41)	162
	Death Pm 30d	-0.0000232	(-0.93)	162
	Death Pm 60d	-0.0000338	(-0.77)	154
	Death Pm 90d	-0.000025	(-0.50)	144
	Death Pm 90d	-9.63E-06	(-0.17)	133
**Hofstede cultural dimensions**	Power distance	13.19	(-1.7)	68
	Individualism	-11.91	(-1.39)	68
	Masc/Fem	8.507	(-1.19)	68
	Uncertainty	6.938	(-0.83)	68
	Long/Short term	-7.125	(-0.89)	90
	Indulgence	-7.454	(-1.02)	91

The cultural dimensions show the following patterns. Smaller values for power distance mean less power distance within society and more egalitarianism. For uncertainty avoidance, scores mean more uncertainty avoidance, i.e. the dominant cultural perspective tends to be discomfort with ambiguity and the preference for avoiding uncertainty. Higher scores indicate more individualism, and lower scores indicate more collectivism. Higher scores indicate a country-wide cultural preference for masculine traits, and lower scores indicate more feminine traits. Finally, higher scores signify more indulgence, and lower scores indicate more restraint.

Next, we compute fractional logit regressions of our outcome variables with robust standard errors. We compute three different models: first, with the covariates excluding the cultural dimension; second, with the covariates including the cultural dimension; and third, with the covariates excluding the cultural dimension alone, but including the cultural dimensions interacted with the indicator for woman leadership. We acknowledge that the small proportion of women-led countries raises questions about the ability to draw conclusions about them in these estimations. We therefore choose our models based on methods designed for such distributions. The interacted model is chosen over split-sample analysis because women-led countries represent less than 10% of the data. Additionally, we use both matching and reweighting schemes to compute the average treatment effect on the treated (ATET), which shows the effect on the proportion on deaths in women lead countries of having a woman leader. We compute the ATET rather than the ATE because there are too few woman-led countries, with too little diversity, to reliably match the full spectrum of male lead countries with female lead countries. Table 5 provides a summary overview of the coefficient signs (+/-) and significance of each of the variables for the full model. All models are provided in full in the S1 Appendix.

**Table 5:** Summary of outcomes from GLM regression (see S1 Appendix for full tables); coefficient signs denoted by (+/-) and significance by (*).

	Case 30d	Case 60d	Case 90d	Case 120d	Death 30d	Death 60d	Death 90d	Death 120d	Death Pm 30d	Death Pm 60d	Death Pm 90d	Death Pm 120d
**Women in Parliament**	-	+	+	+	+•	+•	+	+	+•	+•	+	+
**Free**	+	+	+	+	+	+	+	+	+	+	+	+
**Not free**	+•	+	+	-	+•	+•	+	-	+•	+	+	-
**GDP per capita**	+•	+	+	-	+•	+	-	-	+	+	-	-
**% over 65**	-	-	-	-	-	-	-	-	-	-	-	-
**Land area**	-•	-•	-•	-	-•	-	-	-	-	-	-	-
**Land borders**	+	+	+	+	-	+	+	+•	+	+	+	+•
**Life expectancy**	+	+•	+•	+•	+•	+	+	+•	+	+	+	+
**Power distance**	-•	-	+	+	-	+	+	+	+	+	+	+
**Individualism**	+	+•	+•	+•	+•	+•	+•	+•	+•	+•	+•	+
**Masculine/feminine**	-•	-	-	-	-•	-	-	-	-	-	-	-
**Uncertainty**	+•	+	+	-	+	-	-	-	+	-	-	-
**Long/short term**	-	-•	-•	-	-•	-•	-	-	-•	-	-	-
**Indulgence**	-	-•	-	+	-•	-	+	+	-	-	+	+
**Woman-led country**	-	-•	-	-	-•	-	-•	-	-	-•	-•	-•
**Woman x Power distance**	+•	+	+	+	+	-	+	+	+	+	+	+
**Woman x Individualism**	-•	-	-•	-•	-•	-•	-•	-•	-•	-	-	-
**Woman x Masc/Fem**	-•	-•	-•	-•	-•	-•	-•	-•	-•	-•	-•	-•
**Woman x Uncertainty**	-•	-•	-	-	-•	-	-	-	-•	-	-	-
**Woman x Long/Short term**	+•	+•	+•	+•	+•	+•	+•	+•	+•	+•	+•	+•
**Woman x Indulgence**	+•	+•	+•	+•	+•	+•	+•	+•	+•	+•	+•	+•
**Constant**	-	-•	-•	-•	-•	-•	-•	-•	-•	-•	-•	-•

## Results

### Base model

Tables 1–3 in the S1 Appendix show the base model results for deaths per capita at 30, 60, 90, and 120 days following the first diagnosed case, first death, and first death per million. With only one exception the coefficient on the indicator for woman leadership is negative, but in no case is the coefficient statistically significant. With only one exception, the coefficient on the percentage of women in parliament is positive and it is statistically significant in ten out of twelve models. We consider this strong evidence that countries with high levels of women in parliament have fared worse. One possible explanation for this is in endogenous versus exogenous sources of increased women’s representation in parliaments. In some cases, legislative gender quotas are exogenous to the country’s culture, and often imposed by mediating parties during post-conflict peace settlements. In other cases, countries with greater gender parity in society may endogenously select more women candidates for the legislature because it reflects deep social values about equality and representation.

The coefficient for GDP per capita is positive in all twelve models and statistically significant in five. This is moderately strong evidence that wealthier countries have fared worse than less wealthy countries. The coefficient on the indicator variable for Free is positive in all twelve models and statistically significant in two models. This provides weak evidence that Free countries do worse than Partly Free countries (our base case). The coefficient for Not Free is negative with only one exception and is statistically significant in four of those cases. This is moderately strong evidence that according to the reported figures countries with authoritarian leadership have lower number of COVID deaths. We suggest two potential explanations for this: first, because of the “authoritarian advantage,” Not Free countries may be better able to implement strict lockdown policies that restrict movement or govern social behavior, such as mandating face masks [32]. Second, Not Free countries may also systematically under-count, under-test, or under-report their populations. This may be because of a lack of capacity, or because they have a reputational disincentive to be forthcoming about the depth of the pandemic crisis.

The coefficient for percentage of the population over 65 is positive in seven out of twelve models and statistically significant in none. We do not see any consistency in response to this indicating that life expectancy is capturing the health dimension. Land area is positive in eight out of twelve models but statistically significant in only two models, where it had negative coefficient. There is little evidence that larger countries have fared better or worse than smaller countries. Land borders are positive in all but one model though statistically significant in none. Life expectancy is positive in all twelve models and statistically significant in eight of the models. This is strong evidence that countries with longer life expectancy have fared worse. Fatalities from COVID are concentrated among the elderly and those with significant comorbidities and both these populations are likely larger in countries with longer life expectancies.

We take care to note that the model predicting deaths per capita thirty days after the first case is a clear outlier. We include it in our analyses because the narrative of women’s superior leadership initially took root during this period, corresponding to mid- to late-March subsequent to the World Health Organization declaring Covid-19 a global pandemic. However, thirty days into a pandemic is likely too soon to obtain an accurate picture of countries’ policy responses. Furthermore, many countries changed their metrics, reporting guidelines, and policy interventions with considerable frequency in the early days of the pandemic, creating more uncertainty and instability in the models at thirty days.

### Model with culture

The indicator for woman leadership has a negative coefficient in every model but it is not significant in any model (see Tables 4–6 in the S1 Appendix). The coefficient for power distance is negative in two cases (one of them statistically significant) and positive in ten cases with seven of them statistically significant. Since the one that is negative and statistically significant occurs in our model for the number of deaths 30 days after the first case, our outlier, we consider this strong evidence that increasing power distance increases the number of deaths. The coefficient of Individualism is positive in all twelve models and statistically significant in every model except our outlier. The coefficient for masculinity/femininity is negative in every model but only statistically significant in our outlier. Uncertainty has a positive coefficient in every model but it is significant in only two models, one of which is our outlier. Longterm is positive in ten out of twelve models but not statistically significant in any. The coefficient of Indulgence/Restraint is positive in eight our of twelve models but not statistically significant in any. The cultural dimensions that seem to affect the effectiveness of COVID response are Power Distance and Individualism with increased power distance and increased individualism both increasing reported deaths per capita.

With one exception the coefficient of the percentage of women in parliament is positive and is statistically significant in seven cases. We consider this moderately strong evidence that countries with high levels of women in parliament have fared worse. Interestingly, including the cultural dimensions has changed the coefficients for the Freedom House indicators. Again, the coefficient on the indicator for Free is positive in every case but it is not statistically significant in any. The coefficient for Not Free is now positive in ten of the twelve models and is statistically significant and positive in five. The coefficient for GDP per capita is positive in eight models and negative in four. The coefficient is significant in only 3 models and always with a positive sign. The coefficient for the percentage of the population over 65 is negative in all but our outlier model and is statistically significant with a negative sign in three models. Land area has a negative coefficient in every model but is statistically significant in none. Life expectancy has a positive coefficient in every model but is statistically significant in only one.

### Model with culture interacted with female leadership

To answer the question of how country culture interacts with leader sex, we interact the dichotomous leader variable (0, man; 1, woman) with the six cultural dimensions. We include the un-interacted covariates and the interaction with the indicator for women (see Tables 7–9 in the S1 Appendix). The coefficient therefore represents the additional effect of the variable in countries lead by women. Statistical significance indicates that there is a statistically significant difference in the influence of the variable between countries with women leadership and male leadership.

The measure Power Distance is positive in nine of the eleven models but is statistically significant in none. Female leadership exacerbates the effect of Power Distance with positive coefficients on the interaction in all eleven models. As with Power Distance the coefficient on the interaction is not statistically significant in any model.

The measure Individualism has a positive coefficient in all eleven models and is statistically significant in ten of eleven models. This is very strong evidence that deaths increase with increasing individualism. The effect of female leadership is a moderating effect, with the interaction have a negative coefficient in all eleven models with statistical significance in seven of eleven models. Indeed, the coefficient of individualism for men is positive in all eleven models and statistically significant in ten of eleven. By contrast, for women the coefficient of Individualism is negative in all 11 models and statistically significantly different from 0 in three of the models.

The measure of Masculinity/Femininity has a negative coefficient in every model but it is only statistically significant in one model. Female leadership reinforces the effect of Masculinity/Femininity with statistically significant negative coefficients in all eleven models. Indeed, the coefficient of Masculinity/Femininity is statistically significantly different from 0 for men in only one model but is negative and statistically significant different from 0 for women in all eleven models.

The coefficient of Uncertainty is negative in seven models but not statistically significant in any. The effect of Uncertainty is strengthened for countries with female leader, the coefficient of the interaction is negative in every model but only statistically significant in three. The coefficient for Uncertainty for men is negative in seven models but not statistically significant in any. The coefficient of Uncertainty for countries with female leaders is negative in all eleven models and is statistically significantly different from 0 in one model. We would say that there is little evidence for either an effect of Uncertainty or of disparate effects based on leader gender.

The coefficient of Longterm is negative in all 11 models and statistically significant in five models. The effect is moderated for countries with female leaders, the effect on the interaction is positive and statistically in all eleven models. The coefficient for Longterm for countries with female leaders is positive in all eleven models and statistically significant in ten models. Thus, there is moderate evidence that increased Longterm reduces deaths in countries with male leaders and strong evidence that increased Longterm increases deaths in countries with female leaders.

The coefficient of Indulgence/Restraint is negative in six models and positive in five models, two negative coefficients are significant. The interaction term is positive and statistically significant in every model. This provides strong evidence for a disparate effect depending on leader gender. The coefficient on Indulgence/Restraint for countries with female leaders is positive and statistically significant in all eleven models. Thus there is little evidence of any effect of Indulgence/Restraint in countries with male leaders but strong evidence that increasing Indulgence/Restraint leads to increased deaths in countries with female leaders.

Women in parliament has a positive coefficient in every model and is statistically significant in four of the eleven models. The coefficient for the Free indicator has a positive coefficient in every model but none are statistically significant. The coefficient for the Not Free indicator is positive in eight of eleven models and statistically significant in three of eleven, always with a positive coefficient. GDP per capita has a positive coefficient in six of eleven models and it is statistically significant in only one model. The percentage of the population over 65 has a negative coefficient in every model but it is statistically significant in none. Land area has a negative coefficient in every model and is statistically significant in three. Land borders has a positive coefficient in ten of eleven models but is statistically significant, with a positive coefficient, in only two models. Life Expectancy has a positive coefficient in all eleven models and it is statistically significant in five of the eleven models.

### Nearest neighbor matching

In Model 3 we use nearest neighbor matching (NNM) for the average treatment effect on the treated [ATET] [54, 55]. We implement NNM for the ATET to estimate the effect of having a woman leader on the reported fatality rate per 100,000. There are no significant differences between men and women leaders at 30, 60, or 90 days post first reported case or first reported death. Additionally, some countries drop out of the sample at 90 days post first reported death because the later onset of Covid-19, so the availability of quality matches decreases substantially. Table 6 provides the list of country matches.

**Table 6:** Average treatment effects on the treated nearest neighbor country matches between women-led and men-led countries.

Country	Cases 30 days	Cases 60 days	Cases 90 days	Cases 120 days	Deaths 30 days	Deaths 60 days	Deaths 90 days	Deaths 120 days
**Aruba**								
**Bangladesh**	Bhutan	Bhutan	Bhutan	Bhutan	Morocco	Morocco	Morocco	Morocco
**Barbados**	Uruguay	Uruguay	Uruguay	Uruguay	Uruguay	Uruguay	Uruguay	Uruguay
**Belgium**	Sweden	Sweden	Sweden	Sweden	Sweden	Sweden	Sweden	Sweden
**Bolivia**	Mexico	Mexico	Mexico	Mexico	Mexico	Mexico	Mexico	Mexico
**Denmark**	Netherlands	Netherlands	Netherlands	Netherlands	Netherlands	Netherlands	Netherlands	Netherlands
**Finland**	Sweden	Sweden	Sweden	Sweden	Sweden	Sweden	Sweden	Sweden
**Germany**	Austria	Austria	Austria	Austria	Austria	Austria	Austria	Austria
**Iceland**	United Kingdom	United Kingdom	United Kingdom	United Kingdom	United Kingdom	United Kingdom	United Kingdom	United Kingdom
**Myanmar**	Congo	Congo	Congo	Congo	Congo	Congo	Congo	Congo
**New Zealand**	United Kingdom	United Kingdom	United Kingdom	United Kingdom	United Kingdom	United Kingdom	United Kingdom	United Kingdom
**Norway**	Switzerland	Switzerland	Switzerland	Switzerland	Switzerland	Switzerland	Switzerland	Switzerland
**Serbia**	Montenegro	Montenegro	Montenegro	Montenegro	Montenegro	Montenegro	Montenegro	Montenegro
**Taiwan**								
**Turks and Caicos Islands**								

Because there are so few women countries available overall, and especially at longer time intervals to match in the sample, we explore only the average treatment effect on the treated. To address this aspect, we examine the change in reported fatality rate per 100,000 if women-led countries were instead led by men [56, 57]. Table 7 shows the difference between each woman-led country and a similar male-led country, subtracting the fatality rate of the male-led country from the fatality rate of the woman-led country, and averaging over the sixteen woman-led countries. None of the differences between men and women leaders using the ATET model is statistically significant using this approach either. However, all the coefficient signs are negative, indicating that the directionality of fatalities is negative for countries led by women.

**Table 7:** Average treatment effect on the treated by nearest neighbor matching and inverse probability weighting.

Method	Time	30 Days	60 Days	90 Days	120 days
Average Treatment Effect on the Treated by Inverse Probability Reweighting	Post First Case	0.00000	-0.00002	-0.00005	-0.00007
	Post First Death	0.00000	-0.00004	-0.00007	-0.00008
	Post First One Death per Million	-0.00004	-0.00009	-0.00011	-0.00011
Average Treatment Effect on the Treated by Nearest Neighbor Matching	Post First Case	0.00000	-0.00002	-0.00008	-0.00013
	Post First Death	-0.00001	-0.00009	-0.00013	-0.00015
	Post First One Death per Million	-0.00004	-0.00011	-0.00015	-0.00018

We also use inverse probability weighting since it makes use of the entire spectrum of male countries [58]. We see negligible differences between the coefficients using both the nearest neighbor and inverse probability weighting approaches. Further, coefficient signs are all negative, echoing the findings in both Models 1 and 2.

## Discussion

The key findings from our analysis are as follows: first, across multiple methodological approaches we find no statistically significant difference between the reported Covid-19 fatality rates in countries led by men or women. Free countries tend to have higher reported fatality rates in the month after the first case, but as time progresses the differences between Free, Partly Free, and Not Free become less stark. Wealthier countries, and those with older populations, have more reported fatalities. The length of land borders has no effect on reported Covid-19 fatalities, and land area matters at some points in time, but not others. The role that women in parliament plays is somewhat surprising: more women in the legislature is associated with increased reported Covid-19 fatalities, which we will discuss further below. We summarize our findings in Table 8.

**Table 8:** Summary of hypotheses.

Hypotheses	Outcome
*1*. *Countries led by women chief executives will have fewer Covid-19-related deaths*.	Not supported
*2*. *Countries with stronger feminine culture*, *longer-term orientation*, *more indulgence*, *less power distance*, *greater tolerance for uncertainty*, *and more collectivism will have fewer Covid-19-related fatalities*.	Supported
*3*. *Leadership by a woman will amplify the effects of cultural traits that are already beneficial to pandemic management*, *compared to leadership by a man*.	Supported

Our results suggest that countries led by women are qualitatively different from those led by men, as the double bind and cultural differences theories would predict. From a theoretical perspective, female leaders’ should be ideally positioned to govern better than male leaders through a global pandemic crisis. Female leaders’ focus on communal policies should bolster their countries’ baseline preparedness, while also fulfilling the more masculine agentic frame in their ability to intervene decisively in times of crisis, such as closing borders and mandating quarantine. The underlying cultural values that differentiate countries who have fared better and worse during the pandemic, alongside gendered leadership patterns, will likely matter in the long run in determining how countries and their citizens fare in the post-pandemic landscape.

It may seem puzzling that countries with more women in the legislature fare worse during the pandemic than those with fewer women. However, we note that women’s representation is related to gender quotas [18, 59, 60], which many countries, especially in the developing world, have adopted after ratifying CEDAW [61]. Gender quotas may have counterintuitive effects, especially in Partly Free or Not Free countries [62]. Tripp and Kang [62] suggest that gender quotas may be symbolic, used strategically to obtain women’s votes, or create new patronage networks. The adoption of gender quota legislation by countries may also be a part of a post-conflict peace settlement, or may be used by a country to strategically signal their commitment to democracy. Similar to Collier’s argument about the deleterious effects of rapid democratization—elections without checks and balances [63]–it is likely that rapid or exogenously-influenced adoption of gender quotas may not offer deep social protections in the way that countries with endogenous growth of women in political leadership might have.

Women chief executives have gendered incentives related to the political double bind to attend to both masculine and feminine leadership traits. Women leaders can both care for the national family during a pandemic crisis, while also leading with decisive actions such as closing borders, issuing executive orders, and addressing security-related pandemic concerns. Based on our results, we suggest that public opinion coalesced around the idea that women-led countries were managing the pandemic better following their notable public policy speeches given in mid-March, specifically by the leaders of Germany and New Zealand.

We note several caveats that also likely impact our findings. First, countries led by men far outnumber countries led by women, biasing the sample. We corrected for this using nearest neighbor matching. Second, testing and reporting policies have changed repeatedly within countries in our sample during the reporting time: some countries suffer from well-known under-reporting biases [64], and some countries like Belgium may be over-reporting [65].

Third, women chief executives tend to govern smaller countries, both in terms of geographic and population sizes [8]. While Jacinda Ardern’s leadership has engendered massive public support domestically and abroad for Covid-19 policies related to lockdowns and quarantining, it is comparatively easier to close the borders of a remote island nation than in countries that share multiple, lengthy borders and major international transportation hubs. However, as our land borders variable was not statistically significant, this is clearly not the case everywhere.

Fourth, perceptions of women’s successful leadership may also be a product of Western news bias. Researchers studying the phenomenon of pandemic in outcomes in countries led by men or women have amplified this bias by selecting a subset of cases that demonstrate the expected outcomes [9]. Few news outlets have covered Vietnam’s successes, notably zero Covid-19 fatalities as a result of swift, comprehensive action [66]. It could be that countries with previous epidemic or pandemic experience, like Vietnam, are in a better position to anticipate and respond to the current pandemic. Yet as Bosancianu et al., find, exposure to SARS, MERS, or Ebola does not offer more social protection [67].

Finally, the fatality rate is not the only metric by which a leader or country should be judged. The overall pandemic management strategy, including policies implemented to alleviate suffering and mitigate risk, should be comprehensively evaluated to assess how well a leader or country is faring. While sex or gender may not offer immediate protections against the deadliness of Covid-19, these traits may offer important buffers against downstream problems that could emanate from the effects of the global pandemic, such as social destabilization or contentious political behavior. Women-led countries may fare better in the long run in part due to the strength of institutions, increased trust in government, and decreases in corruption that female leadership engenders.

## Conclusions

This study is the first to comprehensively address the roles of women leaders and women legislators in mitigating the effect of the Covid-19 pandemic. Similar to Bosancianu et al., we find that there are no differences in reported fatalities between women-led and men-led countries [67]. The theory of the political double bind helps explain why women leaders in countries like New Zealand, Iceland, Germany, and Taiwan have garnered ubiquitous praise for their leadership, as they are excelling at deploying both masculine and feminine leadership traits during the pandemic.

However, it may be that we researchers are asking the wrong questions. Women chief executives pursue different policies from their male counterparts, and women in legislatures are less corrupt and more trustworthy, at least optically for constituents [68, 69]. Given the important role of countries’ cultural foundations, perhaps we should also be asking about long-term orientation, societal power disparities, and collectivist policies influence countries’ post-pandemic future trajectories. For example, will countries that have women chief executives and favorable cultural values fare better in the long term while dealing with the aftermath of Covid-19? And, will these countries have lower unemployment numbers, better re-opening plans, and less nationwide and individual economic damage?

It is important to note that while some women chief executives have shown impressive governance during the Covid-19 crisis, this has not translated to statistically significant differences in decreasing the number of cases or deaths in their countries. Early in the pandemic, for example, Belgium—then led by Sophie Wilmès—reported high numbers of Covid-19-related fatalities [65], in part because they were including deaths in nursing homes and suspected deaths as part of their official count. It is possible that this inclusiveness, ensuring that every death counted and mattered, itself was a culturally relevant phenomenon and that leader gender amplified it.

The number of women in parliament did not provide protections either. These results need further disentangling, and suggest that we need to rethink our metrics about what success in the pandemic means, and which exemplars we should be following. Perhaps the economic effect of a pandemic on GDP or other social measures are areas of future research. Too, perhaps the traits of our sample need further analysis. Quiet success stories like Vietnam should be amplified, and we should also continue to investigate the extent to which Covid-19-related interventions have a gendered component. We reiterate that there are only sixteen women chief executives from which to draw conclusions. Evaluating gendered policymaking requires a larger sample of women chief executives. Our results reflect the reality that building resilience is a preparative process, and therefore that pandemic outcomes today depend on policymaking choices made far in advance.

## Supporting information

S1 Appendix (DOCX)
