# Supplementary material for: Gender in the time of COVID-19: Evaluating national leadership and COVID-19 fatalities
Source: PLoS One. 2020 Dec 31;15(12):e0244531. doi: 10.1371/journal.pone.0244531 (PMC7774849; doi:10.1371/journal.pone.0244531)
Supplement: S1 Appendix — (DOCX) [file pone.0244531.s001.docx]

**Appendix**

Table 1. Base model, deaths after first reported cases

|  | Case 30d |  | Case 60d |  | Case 90d |  | Case 120d |  |
| --- | --- | --- | --- | --- | --- | --- | --- | --- |
| Woman | 0.0521 | (0.10) | -0.467 | (-1.07) | -0.359 | (-0.63) | -0.347 | (-0.66) |
| Women in parliament | -0.0223 | (-1.40) | 0.0174 | (1.42) | 0.0384*** | (3.92) | 0.0375*** | (4.36) |
| Free | 1.240** | (2.80) | 0.558 | (1.39) | 0.245 | (0.69) | 0.141 | (0.44) |
| Not Free | 0.0540 | (0.11) | -0.215 | (-0.42) | -0.629 | (-1.45) | -0.864* | (-2.46) |
| GDP per captia | 0.0000103 | (1.30) | 0.0000148** | (2.88) | 0.00000875 | (1.48) | 0.00000454 | (0.78) |
| % over 645 | -0.0373 | (-1.06) | 0.0201 | (0.64) | 0.0221 | (0.73) | -0.0104 | (-0.33) |
| Land area | -0.000000601 | (-1.68) | -0.000000252** | (-2.81) | -2.35e-08 | (-0.46) | 4.42e-08 | (1.02) |
| Land borders | 0.00266 | (0.03) | 0.0398 | (0.65) | 0.0685 | (1.42) | 0.0849 | (1.85) |
| Life expectancy | 0.0682 | (1.79) | 0.0994** | (2.86) | 0.121** | (3.28) | 0.130*** | (3.75) |
| Constant | -18.03*** | (-6.88) | -20.16*** | (-8.25) | -21.32*** | (-8.39) | -20.95*** | (-8.90) |
| N | 167 |  | 167 |  | 167 |  | 166 |  |
| * p<0.05, ** p<0.01, *** p<0.001" | | |  |  |  |  |  |  |

Table 2. Base model, deaths after first reported death

|  | Death 30d |  | Death 60d |  | Death 90d |  | Death 120d |  |
| --- | --- | --- | --- | --- | --- | --- | --- | --- |
| Woman | -0.00478 | (-0.01) | -0.313 | (-0.54) | -0.401 | (-0.72) | -0.390 | (-0.79) |
| Women in parliament | 0.0346** | (2.91) | 0.0427*** | (4.40) | 0.0403*** | (4.65) | 0.0400*** | (4.69) |
| Free | 0.774* | (2.23) | 0.392 | (1.13) | 0.282 | (0.85) | 0.0963 | (0.32) |
| Not Free | 0 | (.) | 0 | (.) | 0 | (.) | 0 | (.) |
| GDP per captia | 0.0000133* | (2.56) | 0.0000131* | (2.41) | 0.00000840 | (1.55) | 0.00000275 | (0.48) |
| % over 645 | 0.0399 | (1.41) | 0.0467 | (1.64) | 0.0131 | (0.45) | -0.0231 | (-0.80) |
| Land area | -0.000000182** | (-2.89) | 1.67e-08 | (0.38) | 5.83e-08 | (1.46) | 6.69e-08 | (1.66) |
| Land borders | -0.0166 | (-0.33) | 0.0255 | (0.55) | 0.0527 | (1.13) | 0.0709 | (1.60) |
| Life expectancy | 0.0444 | (1.15) | 0.0780* | (2.03) | 0.0938** | (2.79) | 0.108*** | (3.44) |
| Constant | -16.89*** | (-5.97) | -18.70*** | (-6.95) | -18.78*** | (-8.19) | -18.79*** | (-8.79) |
| N | 157 |  | 151 |  | 151 |  | 146 |  |
| * p<0.05, ** p<0.01, *** p<0.001" | | |  |  |  |  |  |  |

Table 3. Base model, deaths per million

|  | Death_Pm 30d |  | Death_Pm 60d |  | Death_Pm 90d |  | Death_Pm 120d |  |
| --- | --- | --- | --- | --- | --- | --- | --- | --- |
| Woman | -0.291 | (-0.54) | -0.405 | (-0.71) | -0.428 | (-0.80) | -0.425 | (-0.90) |
| Women in parliament | 0.0377*** | (3.89) | 0.0431*** | (4.85) | 0.0406*** | (4.89) | 0.0416*** | (5.27) |
| Free | 0.563* | (2.09) | 0.284 | (0.95) | 0.184 | (0.64) | 0.122 | (0.45) |
| Not Free | 0 | (.) | 0 | (.) | 0 | (.) | 0 | (.) |
| GDP per captia | 0.0000137** | (2.86) | 0.0000120* | (2.28) | 0.00000627 | (1.18) | 0.000000961 | (0.17) |
| % over 645 | 0.0452 | (1.90) | 0.0314 | (1.18) | -0.00724 | (-0.26) | -0.0448 | (-1.63) |
| Land area | 1.03e-08 | (0.24) | 5.04e-08 | (1.31) | 6.27e-08 | (1.65) | 6.51e-08 | (1.67) |
| Land borders | 0.0369 | (0.89) | 0.0432 | (0.94) | 0.0616 | (1.37) | 0.0859* | (1.96) |
| Life expectancy | 0.0410 | (1.32) | 0.0525 | (1.60) | 0.0762* | (2.51) | 0.0909** | (3.07) |
| Constant | -16.27*** | (-7.33) | -16.14*** | (-7.05) | -16.78*** | (-8.09) | -16.99*** | (-8.35) |
| N | 146 |  | 140 |  | 129 |  | 119 |  |
| * p<0.05, ** p<0.01, *** p<0.001" | | |  |  |  |  |  |  |

Table 4. Model with culture, deaths per capita after first case

|  | Case 30d |  | Case 60d |  | Case 90d |  | Case 120d |  |
| --- | --- | --- | --- | --- | --- | --- | --- | --- |
| Woman | -1.480 | (-1.77) | -0.879 | (-1.66) | -0.616 | (-1.68) | -0.527 | (-1.42) |
| Women in parliament | -0.0101 | (-0.41) | 0.0308 | (1.65) | 0.0459* | (2.39) | 0.0331* | (1.99) |
| Free | -0.687 | (-0.85) | 0.236 | (0.36) | 0.863 | (1.33) | 1.017 | (1.84) |
| Not Free | 1.889** | (2.68) | 1.537* | (2.21) | 0.975 | (1.31) | 0.0257 | (0.04) |
| GDP per capita | 0.0000359** | (3.21) | 0.0000140 | (1.81) | 0.00000496 | (0.59) | -0.00000322 | (-0.37) |
| % over 65 | 0.0583 | (0.78) | -0.0676 | (-1.11) | -0.0940 | (-1.56) | -0.115* | (-2.24) |
| Land area | -0.000000655* | (-2.12) | -0.000000429* | (-2.54) | -0.000000145 | (-1.64) | -5.52e-08 | (-0.85) |
| Land borders | 0.0802 | (0.83) | -0.0179 | (-0.22) | 0.0114 | (0.15) | 0.0721 | (1.19) |
| Life expectancy | -0.0101 | (-0.11) | 0.158* | (2.31) | 0.143* | (2.00) | 0.146* | (2.01) |
| Power distance | -0.0286* | (-1.98) | -0.00256 | (-0.23) | 0.0171* | (1.97) | 0.0164 | (1.91) |
| Individualism | 0.00359 | (0.23) | 0.0325** | (2.74) | 0.0394*** | (4.35) | 0.0314*** | (3.43) |
| Masc/Fem | -0.0324* | (-2.54) | -0.00505 | (-0.56) | -0.00303 | (-0.54) | -0.00609 | (-1.03) |
| Uncertainty | 0.0219* | (2.39) | 0.0166 | (1.82) | 0.0125 | (1.74) | 0.0102 | (1.45) |
| Long/Short term | -0.0278 | (-1.93) | -0.0107 | (-1.00) | 0.000508 | (0.05) | 0.00379 | (0.41) |
| Indulgence | 0.00244 | (0.19) | -0.0108 | (-1.14) | 0.00121 | (0.14) | 0.0132 | (1.36) |
| Constant | -11.28 | (-1.55) | -24.59*** | (-5.30) | -25.31*** | (-5.37) | -24.48*** | (-5.00) |
| N | 60 |  | 60 |  | 60 |  | 60 |  |
| * p<0.05, ** p<0.01, *** p<0.001 | | |  |  |  |  |  |  |

Table 5. Model with culture, deaths per capita after first death

|  | Death 30d |  | Death 60d |  | Death 90d |  | Death 120d |  |
| --- | --- | --- | --- | --- | --- | --- | --- | --- |
| Woman | -0.422 | (-1.20) | -0.567 | (-1.60) | -0.555 | (-1.56) | -0.499 | (-1.35) |
| Women in parliament | 0.0755*** | (4.05) | 0.0593** | (2.85) | 0.0400* | (2.21) | 0.0313 | (1.96) |
| Free | 0.381 | (0.61) | 0.737 | (1.16) | 1.020 | (1.79) | 0.947 | (1.78) |
| Not Free | 2.141** | (3.06) | 1.862* | (2.29) | 0.572 | (0.81) | -0.129 | (-0.21) |
| GDP per capita | 0.0000185** | (3.09) | 0.0000113 | (1.30) | 0.000000784 | (0.09) | -0.00000397 | (-0.43) |
| % over 65 | -0.00964 | (-0.16) | -0.0299 | (-0.48) | -0.0833 | (-1.57) | -0.106* | (-2.12) |
| Land area | -0.000000182* | (-2.28) | -8.28e-08 | (-1.23) | -3.03e-08 | (-0.51) | -8.89e-09 | (-0.16) |
| Land borders | -0.129 | (-1.55) | -0.0433 | (-0.55) | 0.0447 | (0.67) | 0.0840 | (1.50) |
| Life expectancy | 0.0663 | (0.80) | 0.0885 | (1.13) | 0.116 | (1.57) | 0.131 | (1.80) |
| Power distance | 0.0125 | (1.15) | 0.0201* | (2.15) | 0.0184* | (2.11) | 0.0168 | (1.96) |
| Individualism | 0.0286** | (3.22) | 0.0394*** | (4.22) | 0.0331*** | (3.58) | 0.0267** | (2.88) |
| Masc/Fem | -0.00463 | (-0.83) | -0.00107 | (-0.20) | -0.00530 | (-0.90) | -0.00771 | (-1.25) |
| Uncertainty | 0.0197** | (3.05) | 0.00948 | (1.36) | 0.00868 | (1.24) | 0.00930 | (1.34) |
| Long/Short term | 0.00932 | (1.18) | 0.00270 | (0.29) | 0.00503 | (0.55) | 0.00493 | (0.55) |
| Indulgence | -0.00416 | (-0.52) | 0.000570 | (0.06) | 0.0112 | (1.11) | 0.0171 | (1.76) |
| Constant | -22.34*** | (-3.94) | -22.74*** | (-4.40) | -23.13*** | (-4.66) | -23.11*** | (-4.66) |
| N | 60 |  | 59 |  | 59 |  | 59 |  |
| * p<0.05, ** p<0.01, *** p<0.001 | | |  |  |  |  |  |  |

Table 6. Model with culture, deaths per million

|  | Death_Pm 30d |  | Death_Pm 60d |  | Death_Pm 90d |  | Death_Pm 120d |  |
| --- | --- | --- | --- | --- | --- | --- | --- | --- |
| Woman | -0.572 | (-1.79) | -0.583 | (-1.70) | -0.544 | (-1.53) | -0.471 | (-1.22) |
| Women in parliament | 0.0590** | (3.22) | 0.0493* | (2.50) | 0.0354* | (2.13) | 0.0289 | (1.88) |
| Free | 0.508 | (1.12) | 0.752 | (1.41) | 0.978 | (1.76) | 0.947 | (1.68) |
| Not Free | 1.769** | (2.76) | 0.998 | (1.33) | 0.341 | (0.44) | -0.178 | (-0.24) |
| GDP per capita | 0.0000135 | (1.81) | 0.00000647 | (0.75) | -0.00000129 | (-0.15) | -0.00000516 | (-0.56) |
| % over 65 | -0.0169 | (-0.33) | -0.0565 | (-1.02) | -0.0924 | (-1.83) | -0.113* | (-2.18) |
| Land area | -7.63e-08 | (-1.30) | -4.26e-08 | (-0.72) | -2.01e-08 | (-0.37) | -8.14e-09 | (-0.16) |
| Land borders | -0.0298 | (-0.50) | 0.0166 | (0.23) | 0.0621 | (1.05) | 0.0953 | (1.76) |
| Life expectancy | 0.0772 | (1.02) | 0.0865 | (1.16) | 0.104 | (1.37) | 0.122 | (1.67) |
| Power distance | 0.0200** | (2.62) | 0.0198* | (2.35) | 0.0179* | (2.07) | 0.0159 | (1.84) |
| Individualism | 0.0395*** | (5.05) | 0.0381*** | (4.31) | 0.0301*** | (3.40) | 0.0237** | (2.61) |
| Masc/Fem | -0.000817 | (-0.17) | -0.00267 | (-0.49) | -0.00573 | (-0.95) | -0.00758 | (-1.23) |
| Uncertainty | 0.0111 | (1.76) | 0.00901 | (1.33) | 0.00903 | (1.34) | 0.00927 | (1.35) |
| Long/Short term | 0.000766 | (0.10) | 0.00334 | (0.36) | 0.00516 | (0.58) | 0.00340 | (0.38) |
| Indulgence | -0.00147 | (-0.18) | 0.00580 | (0.60) | 0.0160 | (1.49) | 0.0189 | (1.85) |
| Constant | -22.44*** | (-4.55) | -21.71*** | (-4.40) | -21.75*** | (-4.32) | -21.93*** | (-4.52) |
| N | 58 |  | 58 |  | 57 |  | 57 |  |
| * p<0.05, ** p<0.01, *** p<0.001 | | |  |  |  |  |  |  |

Table 7. Full model: Generalized Linear Model for number of reported deaths from the first reported cases at 30, 60, 90, and 120 days

|  | Case 30d |  | Case 60d |  | Case 90d |  | Case 120d |  |
| --- | --- | --- | --- | --- | --- | --- | --- | --- |
| Women in parliament | -0.004 | -0.160 | 0.019 | -1.010 | 0.037 | -1.760 | 0.028 | -1.570 |
| Free | 0.056 | -0.070 | 0.439 | -0.590 | 0.996 | -1.420 | 1.003 | -1.780 |
| Not free | 2.541*** | -3.780 | 1.364 | -1.900 | 0.681 | -0.880 | -0.179 | -0.300 |
| GDP per capita | 0.0000292** | -2.900 | 0.000 | -1.230 | 0.000 | -0.150 | 0.000 | -0.570 |
| % over 65 | -0.014 | -0.170 | -0.086 | -1.360 | -0.089 | -1.460 | -0.097 | -1.770 |
| Land area | -0.000000578** | -2.760 | -0.000000416** | -2.710 | -0.000000180* | -1.990 | 0.000 | -1.200 |
| Land borders | 0.003 | -0.030 | 0.026 | -0.300 | 0.075 | -0.890 | 0.123 | -1.840 |
| Life expectancy | 0.043 | -0.440 | 0.258** | -3.220 | 0.211** | -2.630 | 0.187* | -2.360 |
| Power distance | -0.0405** | -3.100 | -0.021 | -1.670 | 0.005 | -0.690 | 0.008 | -0.940 |
| Individualism | 0.008 | -0.470 | 0.0274* | -2.080 | 0.0320*** | -3.520 | 0.0246** | -2.840 |
| Masc/Fem | -0.0340* | -2.450 | -0.011 | -1.110 | -0.006 | -1.120 | -0.008 | -1.230 |
| Uncertainty | 0.0287* | -2.500 | 0.013 | -1.230 | 0.001 | -0.120 | -0.001 | -0.100 |
| Long/Short term | -0.025 | -1.830 | -0.0207** | -2.700 | -0.0175* | -2.370 | -0.013 | -1.640 |
| Indulgence | -0.001 | -0.060 | -0.0214* | -2.270 | -0.008 | -0.890 | 0.006 | -0.660 |
| Woman | -2.827 | -0.580 | -8.185* | -2.250 | -4.220 | -1.920 | -4.187 | -1.940 |
| Woman x power distance | 0.224** | -2.970 | 0.123 | -1.770 | 0.016 | -0.520 | 0.008 | -0.200 |
| Woman x individualism | -0.368*** | -3.370 | -0.125 | -1.340 | -0.163** | -2.690 | -0.144* | -2.240 |
| Woman x masc/fem | -0.358*** | -10.150 | -0.0720* | -2.000 | -0.0607*** | -4.020 | -0.0533** | -2.860 |
| Woman x uncertainty | -0.155*** | -4.700 | -0.0655* | -1.980 | -0.028 | -1.960 | -0.018 | -0.900 |
| Woman x long/short term | 0.302*** | -5.780 | 0.123* | -2.040 | 0.165*** | -5.130 | 0.148*** | -4.370 |
| Woman x indulgence | 0.338*** | -5.630 | 0.184** | -3.090 | 0.154*** | -3.730 | 0.139*** | -3.320 |
| Constant | -14.680 | -1.910 | -29.25*** | -5.360 | -27.41*** | -5.210 | -25.14*** | -4.690 |
| N | 60.000 |  | 60.000 |  | 60.000 |  | 60.000 |  |
| * p<0.05, ** p<0.01, *** p<0.001 | |  |  |  |  |  |  |  |

Table 8. Full model: Generalized Linear Model for number of reported deaths from the first reported deaths at 30, 60, 90, and 120 days

|  | Death 30d |  | Death 60d |  | Death 90d |  | Death 120d |  |
| --- | --- | --- | --- | --- | --- | --- | --- | --- |
| Women in parliament | 0.0586*** | -3.31 | 0.0490* | -2.3 | 0.0331 | -1.7 | 0.0274 | -1.63 |
| Free | 0.524 | -0.76 | 0.854 | -1.26 | 0.981 | -1.65 | 0.908 | -1.69 |
| Not free | 1.880** | -2.83 | 1.660* | -2.06 | 0.381 | -0.53 | -0.233 | -0.39 |
| GDP per capita | 0.0000130* | -2.12 | 0.00000822 | -1 | -0.00000054 | -0.06 | -0.00000463 | -0.46 |
| % over 65 | -0.033 | -0.5 | -0.0162 | -0.23 | -0.0609 | -1.05 | -0.0846 | -1.56 |
| Land area | -0.000000224* | -2.46 | -0.000000115 | -1.64 | -5.39E-08 | -0.85 | -2.65E-08 | -0.43 |
| Land borders | -0.0642 | -0.83 | 0.025 | -0.31 | 0.102 | -1.38 | 0.130* | -2.15 |
| Life expectancy | 0.176* | -2.06 | 0.147 | -1.69 | 0.155 | -1.91 | 0.157* | -2 |
| Power distance | -0.00833 | -0.65 | 0.00736 | -0.97 | 0.00943 | -1.17 | 0.00917 | -1.1 |
| Individualism | 0.0212* | -2.41 | 0.0302*** | -3.51 | 0.0254** | -2.95 | 0.0201* | -2.28 |
| Masc/Fem | -0.00958* | -1.96 | -0.00388 | -0.76 | -0.0071 | -1.17 | -0.00909 | -1.34 |
| Uncertainty | 0.00956 | -1.33 | -0.00326 | -0.51 | -0.00265 | -0.41 | -0.000498 | -0.08 |
| Long/Short term | -0.0121* | -2.39 | -0.0179** | -2.58 | -0.0128 | -1.59 | -0.0106 | -1.25 |
| Indulgence | -0.0179* | -2.3 | -0.00949 | -1.09 | 0.0034 | -0.34 | 0.0113 | -1.17 |
| Woman | -4.392* | -2.17 | -4.15 | -1.94 | -4.433* | -2.06 | -4.152 | -1.91 |
| Woman x power distance | 0.027 | -0.8 | -0.000945 | -0.04 | 0.00446 | -0.13 | 0.0115 | -0.31 |
| Woman x individualism | -0.159* | -2.37 | -0.159** | -2.68 | -0.140* | -2.22 | -0.132* | -2.02 |
| Woman x masc/fem | -0.0429* | -2.49 | -0.0567*** | -4.11 | -0.0516** | -2.86 | -0.0492* | -2.5 |
| Woman x uncertainty | -0.0391* | -2.5 | -0.0119 | -0.86 | -0.0138 | -0.72 | -0.0196 | -1.03 |
| Woman x long/short term | 0.158*** | -4.11 | 0.162*** | -5.1 | 0.146*** | -4.31 | 0.140*** | -4.02 |
| Woman x indulgence | 0.158*** | -3.65 | 0.145*** | -3.56 | 0.136** | -3.2 | 0.129** | -3.14 |
| Constant | -26.10*** | -4.38 | -23.83*** | -4.11 | -23.45*** | -4.28 | -22.97*** | -4.25 |
| N | 60 |  | 59 |  | 59 |  | 59 |  |
| * p<0.05, ** p<0.01, *** p<0.001 | |  |  |  |  |  |  |  |

Table 9. Full model: Generalized Linear Model for number of reported deaths from the first reported deaths at 30, 60, 90, and 120 days per one million

|  | Death_Pm 30d |  | Death_Pm 60d |  | Death_Pm 90d |  | Death_Pm 120d | |
| --- | --- | --- | --- | --- | --- | --- | --- | --- |
| Women in parliament | 0.0530** | -2.93 | 0.0433* | -2.05 | 0.0308 | -1.8 | 0.0259 | -1.72 |
| Free | 0.619 | -1.36 | 0.736 | -1.3 | 0.837 | -1.53 | 0.833 | -1.53 |
| Not free | 1.726** | -2.8 | 0.856 | -1.09 | 0.0761 | -0.1 | -0.391 | -0.55 |
| GDP per capita | 0.0000122 | -1.75 | 0.00000556 | -0.67 | -0.00000211 | -0.23 | -0.00000569 | -0.55 |
| % over 65 | -0.00445 | -0.07 | -0.0321 | -0.53 | -0.0679 | -1.22 | -0.0897 | -1.61 |
| Land area | -0.000000101 | -1.62 | -6.57E-08 | -1.08 | -3.56E-08 | -0.61 | -1.91E-08 | -0.33 |
| Land borders | 0.0219 | -0.33 | 0.0747 | -0.93 | 0.113 | -1.71 | 0.141* | -2.45 |
| Life expectancy | 0.119 | -1.49 | 0.121 | -1.51 | 0.134 | -1.65 | 0.145 | -1.85 |
| Power distance | 0.0106 | -1.94 | 0.0111 | -1.5 | 0.00979 | -1.18 | 0.00817 | -0.96 |
| Individualism | 0.0325*** | -4.5 | 0.0301*** | -3.58 | 0.0229** | -2.73 | 0.017 | -1.93 |
| Masc/Fem | -0.00333 | -0.73 | -0.00458 | -0.84 | -0.00733 | -1.13 | -0.00927 | -1.31 |
| Uncertainty | 0.00107 | -0.18 | -0.00221 | -0.34 | -0.00108 | -0.17 | -0.000418 | -0.07 |
| Long/Short term | -0.0165* | -2.38 | -0.0149 | -1.78 | -0.0109 | -1.33 | -0.0116 | -1.39 |
| Indulgence | -0.00937 | -1.2 | -0.00173 | -0.18 | 0.00919 | -0.88 | 0.0133 | -1.31 |
| Woman | -3.54 | -1.81 | -4.452* | -2.06 | -4.662* | -2.07 | -4.620* | -2.03 |
| Woman x power distance | 0.0182 | -0.65 | 0.0258 | -0.67 | 0.029 | -0.67 | 0.0253 | -0.63 |
| Woman x individualism | -0.118* | -2.07 | -0.102 | -1.5 | -0.0935 | -1.32 | -0.109 | -1.57 |
| Woman x masc/fem | -0.0369** | -2.68 | -0.0406* | -2.18 | -0.0404* | -2.01 | -0.0437* | -2.13 |
| Woman x uncertainty | -0.0301* | -2.51 | -0.0287 | -1.34 | -0.0304 | -1.24 | -0.027 | -1.31 |
| Woman x long/short term | 0.129*** | -4.22 | 0.127*** | -3.54 | 0.123*** | -3.38 | 0.130*** | -3.62 |
| Woman x indulgence | 0.110** | -2.97 | 0.103* | -2.19 | 0.0999* | -2.08 | 0.113* | -2.54 |
| Constant | -23.05*** | -4.3 | -21.79*** | -4.07 | -21.67*** | -4.01 | -21.54*** | -4.1 |
| N | 58 |  | 58 |  | 57 |  | 57 |  |
| * p<0.05, ** p<0.01, *** p<0.001 | |  |  |  |  |  |  |  |

Table X. Countries excluded from the analysis

| Men-led | Afghanistan Albania Algeria Angola Antigua and Barbuda Armenia Azerbaijan Bahamas Bahrain Belarus Belize Benin Bhutan Bosnia and Herzegovina Botswana Brunei Burkina Faso Burundi Cambodia Cameroon Central African Republic Chad Comoros Congo Costa Rica Cote d'Ivoire Cyprus Democratic Republic of Congo Djibouti Dominican Republic Ecuador Egypt Equatorial Guinea Eritrea Ethiopia Fiji Gabon Gambia Georgia Ghana Grenada Guatemala Guinea Guinea-Bissau Guyana Haiti Honduras Iraq Israel Jamaica Jordan Kazakhstan Kenya Kuwait Kyrgyzstan Laos Lebanon Lesotho Liberia Libya Madagascar Malawi Maldives Mali Mauritania Mauritius Moldova Mongolia Montenegro Mozambique Namibia Nepal Nicaragua Niger Nigeria Oman Panama Papua New Guinea Paraguay Qatar Rwanda Saint Lucia Saint Vincent and the Grenadines Sao Tome and Principe Saudi Arabia Senegal Seychelles Sierra Leone South Africa Sri Lanka Suriname Tajikistan Tanzania Thailand Timor Togo Tunisia Uganda Ukraine United Arab Emirates Uzbekistan Venezuela Vietnam Yemen Zambia Zimbabwe |
| --- | --- |
| Women-led | Barbados Bolivia Iceland Myanmar |
